# Urinary concerns among older adults: a qualitative analysis in the context of healthy aging

**DOI:** 10.1186/s12877-024-05191-y

**Published:** 2024-07-15

**Authors:** Shaoqing Ge, Kuan-Ching Wu, Shao-Yun Chien, Xianglan Jin, Suah Park, Basia Belza

**Affiliations:** 1 School of Nursing, University of Texas at Austin, Austin, TX USA; 2 School of Nursing, University of Washington, Seattle, WA USA; 3 de Tornyay Center for Healthy Aging, University of Washington School of Nursing, Seattle, WA USA

**Keywords:** Independent living, Urinary concerns, Management, Nursing, Strategy

## Abstract

**Background:**

Urinary concerns increase with age impacting health and quality of life. The aims of this study were to describe: (1) urinary concerns as an age-related change (ARC); (2) the challenges of urinary concerns; (3) adaptation strategies used to manage urinary concerns; and (4) the value of engaging with aging (EWA) as a framework to promote self-management of urinary concerns.

**Methods:**

Data was used from semi-structured interviews with 29 older adults (mean age 77 years). An iterative coding process was used. A codebook was developed based on a-priori themes derived from the EWA framework, our previous publication, and a line-by-line coding of one of the transcripts. As the analysis progressed, additional codes emerged, enriching the codebook.

**Results:**

Six themes emerged: (1) the participants’ experiences; (2) responses to urinary concerns, (3) adaptation and management strategies; (4) knowledge and understanding of urinary concerns; (5) available capacities and resources; and (6) the impact of the COVID-19 pandemic on urinary concerns. Participants tended to address their urinary concerns by adjusting routines, medication schedules, or diet patterns. They tried to secure restroom locations or use tools or reminders to resolve their urinary concerns. COVID-19 led to increased inconvenience for older adults to engage in outdoor activities due to the closure of public restrooms.

**Conclusions:**

Our in-depth qualitative analysis found that participants developed personalized adjustments to address their needs and abilities to their urinary concerns. These findings offer insights into the individual aging experience, which will further enhance our understanding and advancement of person-centered care.

## Background

Throughout the world, the population of older adults is increasing. According to the United Nations [1], 1 in 6 individuals will be aged 65 or older by 2050, and according to the U.S. Census Bureau [2], those in the U.S. aged 65 and older will nearly double from 52 million in 2018 to 95 million by 2060, constituting 23% of the total U.S. population. It is therefore critically important for policymakers, researchers, and clinicians to focus on the health and well-being of older adults.

As older adults age, they experience common age-related changes and chronic conditions. Age-related changes are natural progressive maturational developments accompanied by physical and cognitive alterations associated with aging [3, 4]. Urinary concerns, including urinary incontinence and other related urinary issues, are one category of such changes highly prevalent among older adults. Urinary concerns impact an individual’s quality of life, often not only with physical but with psychological and social consequences [5]. These include embarrassment, reduced mobility, social isolation, and an increased risk of falls and fractures [6]. Moreover, the economic burden of urinary concerns is substantial: high healthcare costs are associated with their diagnosis, management, and treatment [7]. The etiology of urinary concerns among older adults is multifactorial, encompassing a complex interplay of physiological, psychological, and environmental factors [5]: age-related changes in the urinary system, comorbid medical conditions, medications, cognitive impairment, and functional decline. Yet older adults who successfully manage urinary concerns have the potential to improve their overall well-being as a step in healthy aging and the enhancement of quality of life [8–11]. Understanding age-related changes and older adults’ adaptations to them from their own perspective can inform the development of person-centered, targeted programs and services to address their urinary concerns.

**Fig. 1:**
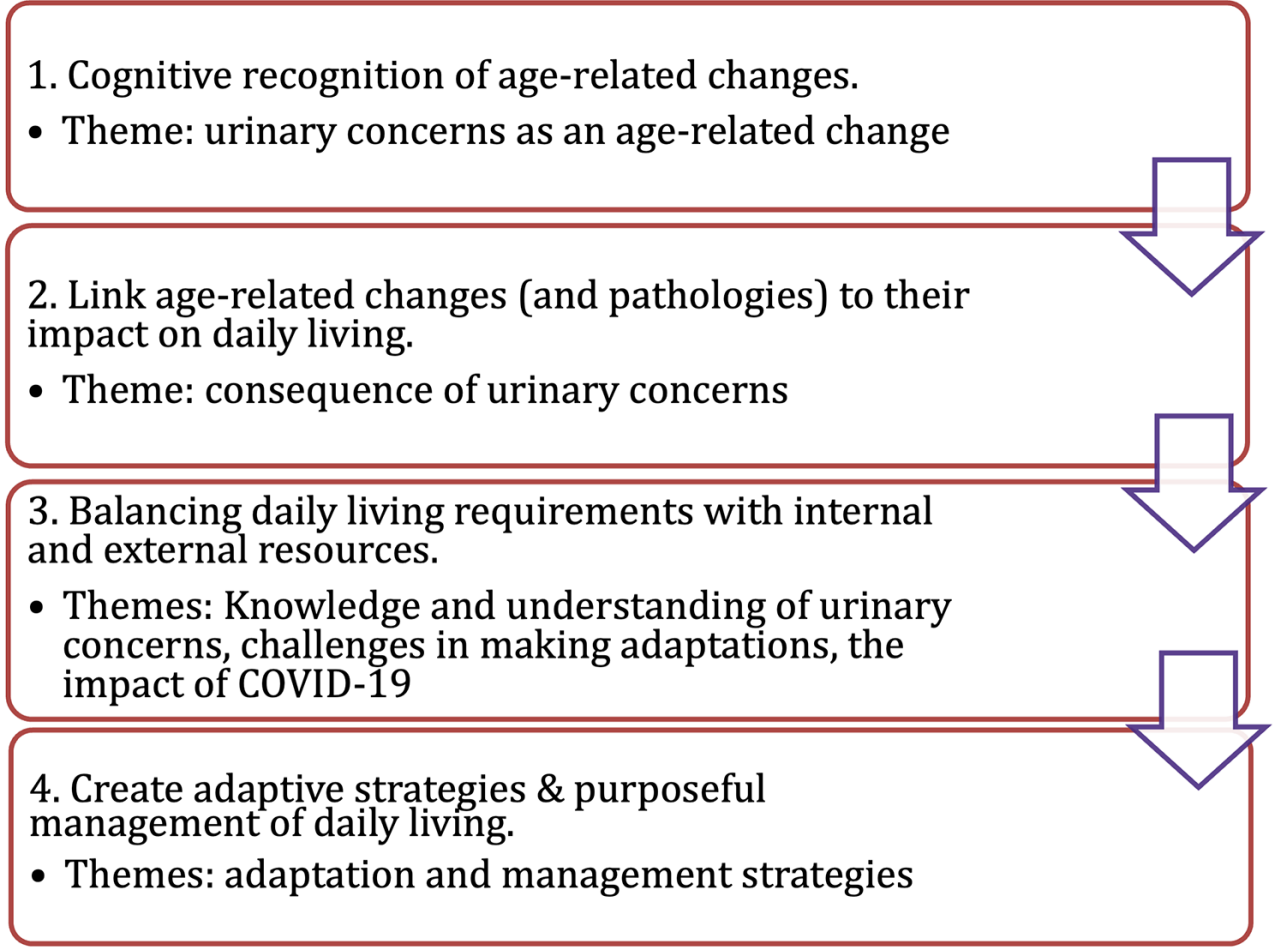
A graphic demonstration of the Conceptual Framework of Engaging with Aging. Revised based on Carnevali, Primomo, & Belza (2019) and the Associated Themes in the Current Study

The conceptual framework of Engaging with Aging (EWA) [3] offers an opportunity to study age-related changes among older adults (see Fig. 1). Previously, EWA was systematically developed based on Ms. Carnevali’s professional nursing knowledge as well as her firsthand aging experience as a centenarian [3, 12], and backed up by the themes generated from her blog postings [11]. EWA provides a perspective on the aging process as an active, conscious effort by older adults to manage age-related changes such as urinary concerns in their daily lives [3, 12]. Despite the value of EWA in studying the daily lives of older adults, only one empirical study has been conducted using EWA as the guiding framework [13]. Specifically, EWA was used to describe the patterns of how older adults adapt to various ARCs in general, but has not been used to focus on any specific type of ARC such as urinary concerns. On the basis of first-hand information provided by older adults themselves, EWA can enable researchers to understand processes with which older adults may proactively and continuously manage the gradual progression of urinary concerns, as well as their psychosocial aspects, that influence their daily lives [3, 12–15].

In this study, we employ EWA as our guiding framework to (1) describe the phenomenon of urinary concerns as an age-related change, (2) identify adaptive strategies used by older adults to manage urinary concerns, and (3) describe additional challenges of urinary concerns caused by contextual shifts such as the COVID-19 pandemic.

## Methods

With EWA as our conceptual framework, we used directed qualitative content analysis with iterative coding of interviews to describe urinary concerns and adaptations among older adults.

### Participants and setting

In a primary study, we conducted semi-structured interviews with 29 older adults in order to describe and understand age-related changes and corresponding adaptations [13]. The interviews were conducted remotely during the COVID-19 pandemic. Participants were recruited through convenience and snowball sampling in communities in the greater Seattle and Puget Sound area in the state of Washington. Inclusion criteria were as follows: age 60 and older, independence in performing activities of daily living, ability to read and speak English, and access to the internet. Details of participants’ characteristics and recruitment strategies can be found in our prior publication [13]. Urinary concerns were mentioned in 15 of 29 interviews. Those interviews are the focus of this paper.

### Data collection

Data were collected in semi-structured one-on-one online interviews from November 2020 through February 2021. Trained team members, who included undergraduate and doctoral nursing students and faculty members, conducted the interviews with an interview guide developed on the basis of findings from a pilot study. The interviews lasted 75 to 100 min, and they were audio recorded and uploaded to a secure online platform.

### Data analysis

Verbatim transcriptions of the interviews were imported into ATLAS.ti 23 software to aid qualitative analysis. As the first step of content analysis [16, 17], we created an initial codebook based on *a priori* themes derived from the EWA framework [3], prior analysis of the EWA blog [11], a previous publication [13], and initial codes of a sample interview transcript that all team members coded independently. All team members reviewed annotations, reconciled coding discrepancies, identified themes, and finalized an initial version of the codebook. Then, four team members paired up as two dyads (K.W. and S.C.; X.J. and S.P. ), and each dyad coded approximately half of the remaining transcripts. Each team member coded the transcripts individually and compared them within each dyad to ensure reliability between coders. When data did not fit existing codes, new codes were added to the codebook [17]. Any disagreements in coding were resolved through discussion and review of the transcripts. The first author (S.G.) also reviewed the entire coding process and facilitated reconciliation when two members in a dyad did not achieve consensus.

Data analysis included multiple strategies to maintain rigor and trustworthiness [18]. First, when we created the initial codebook and added emerging codes, we maintained a level of abstraction by keeping the number of concepts within reasonable limits—6 themes, with 1 to 8 subthemes. Otherwise, a large number of concepts could indicate incomplete or overlapping categorization. The codebook, along with detailed decision logs, was shared among all team members. Second, rather than work alone, we paired up and double-coded each transcript when we analyzed and interpreted the data, which helped to ensure thorough comprehension and sound interpretation [19]. Third, we tracked consistency in coding between coders. We examined each subsequent transcript after confirming minimal deviation in the dyad coding of each preceding transcript and reaching complete consensus, because high intercoder reliability is required in deductive data analysis with multiple coders [20]. Last, all team members met frequently to discuss their thoughts and opinions concerning coding and categorization, to enhance trustworthiness [18]. In addition to weekly team meetings, each dyad met regularly to check the data interpretation’s adequacy and the coding’s consistency.

## Results

### Common symptoms of urinary concerns

Among the fifteen participants whose interviews were analyzed in the current study, ten were females and five were males, with a mean age of 76.8 years old. Participants described four urinary symptoms. The symptom mentioned most often was urinary frequency (mentioned by 8 participants). Thus, as one of the participants said, “So what I’ve noticed, and this has only been happening for maybe the last three months is that I seem to have to urinate more often” (P10). Urgency was reported by 5 participants: “I have more urgency to go to the bathroom, but I don’t lose control” (P08). Polyuria was mentioned by 4 participants: “Now, it’s probably more like an hour. But my bladder is full, it’s not like I just have these little trickles, I have a full bladder that has to be emptied” (P10). Four participants mentioned leakage and/or incontinence: “It becomes somewhat incontinent” (P13).

### Factors contributing to urinary concerns

Six participants attributed the underlying causes of their urinary concerns to diseases or medications. Thus 3 male participants specifically mentioned benign prostatic hyperplasia as the cause of their urinary concerns: “As you age, your prostate starts growing and then it squeezes. Makes you feel like you have to go take a pee more often than you did before. It’s aging. It’s an aging process. Your prostate grows; and, like I said, they can go in there and they can ream it out, which they did mine. Some people it works great on, some people your prostate grows back” (P19).

Unwanted side effects of medications included those of diuretics: “I take a diuretic (for atrial fibrillation), and that inclines one to have some of the symptoms (urinary symptoms) … that I would not have regularly” (P06).

Six participants considered their urinary concerns to be a consequence of the natural process of aging and viewed them as typical signs of aging. However, 2 participants did express uncertainty regarding the underlying causes of their urinary concerns.

### Emotional responses to urinary concerns

Initial emotional responses to urinary concerns tended to be negative. Participants reported feeling upset and/or frustrated when they first encountered this issue, emotional responses included shame, horror, and unhappiness.

However, emotions and attitudes toward urinary concerns appeared to undergo a shift away from the initial negative emotions over time. Some individuals adapted and became more accepting of their condition, and some even found humor in it. One participant said that “The joke of the day was how many times in the night did you have to get up and go pee? You keep track of those things” (P19). In addition, these older adults tended to adopt a more relaxed approach if they knew peers who experienced similar situations: “It’s not particularly embarrassing, because it’s happening to all of my friends” (P08). Some, however, still found their condition to be an annoyance.

### Consequences of urinary concern

The consequences of urinary concerns exerted a substantial influence on participants’ daily lives and activities. Participants said that they had to pay attention to their fluid intake patterns and make changes: “I have to be careful of the amount of liquid I intake I have before going to the grocery, or going out shopping, or … going on a trip, or whatever I’m doing. I just have to be careful of my liquid intake” (P19). This interference with daily living necessitated adaptations and adaptive strategies. As one participant said, “Sometimes I think that’s why I don’t want to drink water because I am thinking, if I drink water then 20 minutes later, I’m going to have to get up and go to the bathroom, which is usually what happens” (P22).

Sleep disturbances also emerged as a prominent consequence related to changes in urinary functions: “Changes in urinary function and changes in sleep sort of go together because I find that I have to get up in the night a couple of times to urinate” (P20). In addition, 2 participants specifically highlighted that urinary concerns induced mental stress.

### Adaptations and management of urinary concern

The adjustment of dietary patterns and food options was a prevalent strategy in managing urinary concerns: “The thing I realize is for heaven’s sake, don’t use carbs and don’t drink. I don’t drink carbonated beverages … period. That’s another thing I don’t do. And that has made a huge difference for me” (P20).

Changes in daily routines were mentioned approximately 13 times. For example: “When I go out, I have to make sure that I’ve used the bathroom on the way out the door. Whether I think I have to or not, I try to use the bathroom. I plan to be out less time unless I’m going to somebody’s house, which I don’t do much of these days. You have to think about it more” (P23). Participants also indicated seeking healthcare—for example, by consulting a doctor—in 6 instances. Some participants had regular annual check-ups with healthcare professionals: “Well, I see a urologist once a year just to have a check-up” (P19). Adjusting medication schedules was reported 6 times: “I said, you take the pill early when I first get up, which is what I did this morning, and then don’t have liquids after late in the evening and then it’s not as much of a problem” (P20). The use of medications and/or supplements, as well as that of pads, were each mentioned 2 times. Exercise was referenced 3 times; scheduling a routine or strategy, 7 times; reminders, 4 times; and employing various tools, technologies, or games as potential management strategies, 6 times.

### Sources of knowledge and information regarding urinary concerns

Participants accessed diverse sources of knowledge and information about urinary concerns. Some, especially those with backgrounds in healthcare, drew from personal experiences: “Yeah, I’m familiar with these things. I worked in a medical doctor’s office for a while … I’m familiar with this when you asked, Do I understand it or whatever? I understand that it exists and there are many reasons why” (P06).

Discussions and conversations with family members and friends also served as valuable sources of information: “My father was talking about it for about 30 years, and my grandfather was talking about it. They were talking about it and then as I got older, my friends started talking about having this issue” (P19). “Over the years, I’ve been engaged in male banter with friends about these kinds of issues. One of the people we travel with has it much more severe than I do. He always has to go where there’s a bathroom. He had chatted about it” (P26). But the same participant also added that “I would not ask him for advice.”

After onset of their condition, participants actively sought knowledge: “Well, gathering information, understanding the problem and how to deal with it makes me feel I can deal with it” (P13). The internet was a main resource: “I looked up some stuff online … It was a good education outside of the doctor’s office, just for my information about what’s going on as it relates to age. And that’s I think the beauty of the internet. You can look up things and not all of it is credible, but you have to think about what you’re researching and get educated and not let your doctor be your only source of knowledge. I mean, that’s what’s nice about the internet, you can get from different sources. Whether it’s the Mayo Clinic or the WebMD, I think that’s a pretty common medical site” (P22). Participants also sought literature to gain insights, such as those provided by common recommendations in research: “This is a common problem for senior men and if you read all the literature about that thing, it will tell you to stop drinking in the evening and stopped drinking carbonated and caffeinated beverages. It’s not rocket science that’s for sure” (P20).

### Attitudes toward adaptations

Six participants expressed high satisfaction with their current adaptations, assigning scores ranging from 8 to 10, resulting in a total of 28 relevant codes in the content analysis. As one participant confidently stated, “I’ll be a 10 (out of 10) as for satisfaction with that” (P08). Some participants had gradually accepted their urinary concerns over time, feeling in control of their situation and appreciating their good health: “I feel like I’m in control of what’s going on. I feel very grateful for having good health and I’m very proactive in wanting to stay that way because there are other friends that I know who have dealt with really tough things” (P22).

Others felt confident in their ability to handle related symptoms, alleviating their worries: “Well, I just feel I know what’s going on and I know how to deal with it. And so I’m feeling positive about my ability to handle it” (P13). Another spoke similarly: “I’ve learned what to do and I think it’s working. I believe that those solutions have worked” (P20). On the other hand, some recognized room for improvement in their adaptation strategies. Participant 12 rated his satisfaction with his adaptation strategy as 6 out of 10: “I’d say six with that. Yes. Eight with getting the information, six with the follow through.”

A total of 18 relevant codes indicated that urinary concerns had minimal or no impact on the participants: the statement that “it’s been with me a very long time and it doesn’t bother me much” (P14) represented the long-term nature of the condition without significant disruption, and another participant (P07) described the impact similarly as “very slight,” noting occasional urges to use the restroom but with minimal disturbance to daily activities.

Conversely, some participants were dissatisfied or disappointed with previous treatments, and this affected their attitudes toward adaptations: “Because it’s such a common problem, I’ve asked people, and there has not been any good resolution to trying any serious techniques and methods. I tried being operated on, which was a fairly not easy operation with a sling, and it didn’t work. I was told that by one of the doctors at the clinic that I went to. Yeah, that’s why my attitude is such” (P06).

### Challenges to adaptations

Participants faced challenges in making adaptations. Four had been unwilling to ask for help when they tried to adapt. Participants hesitated to seek help from others proactively, because they believed that urinary concerns were a personal issue that they preferred to handle on their own: “Oh, this has all been a very personal thing that I’ve handled myself, and I don’t have to get any help from anyone” (P06).

Meanwhile, some who sought help found a lack of support from others; significant others, for example, might refuse help or be unable to offer any suggestions: “My wife is a physician, a retired physician, I might ask her for advice, although she would immediately say to me, ‘That’s not my organ and don’t ask me those questions. You go see a real doctor’” (P26).

Another challenge lay in the time-consuming process of finding suitable adaptation methods: “No, it took me a long time before I finally realized that I simply had to stop drinking carbonated beverages” (P20). The pandemic also imposed constraints on adaptations, primarily because many public facilities were closed, which affected participants’ access to restrooms: “I do a lot of walking, but now I have to think about whether there’s a bathroom on the walk and the pandemic hasn’t helped. All public restrooms are closed” (P26).

### Results of adaptation

Over time, participants agreed that their adaptations were meeting their needs and that they found their urinary concerns increasingly manageable, attributing their ease to effective strategies. One thus described the convenience of an adaptive ritual: “Oh, the ritual is easier because they’re wrapped singly and put into a large plastic bag, and then every few days I take it down and put it down the shoot. I’ve become accustomed to managing it well” (P06). Another outlined an adapted routine, emphasizing the timing of medication intake and liquid consumption: “I said, you take the pill early when I first get up, which is what I did this morning, and then don’t have liquids after late in the evening, and then it’s not as much of a problem” (P20).

Some participants expressed uncertainty about the effectiveness of their adaptations. The following comment exemplifies uncertainty about the efficacy of taking an over-the-counter medication intended to enhance urinary function: “Then, I take an over-the-counter pill that’s supposed to help with urinary function. Slow it down. Make it better. Or, again, I don’t know whether it helps or not. I do it anyway … I don’t know if that helped or not, but I had it done” (P19). Thus participants were willing to make adaptations even when they were uncertain about their strategies’ effectiveness, offering an additional perspective of how the elderly population engages with aging in addressing urinary concerns.

## Discussion

In this study, we have used EWA as a framework to understand urinary concerns as an age-related change in older adults. We have explored how older adults adapt to their urinary concerns, and we have examined challenges that they face in the context of aging during a pandemic. Urinary concerns were prevalent among these older adults, who were often capable of coming up with strategies to manage their symptoms. Consequences included inconveniences in daily life, with sleep disturbances being notable. Participants were able to develop strategies for adaptation on their own and needed minimal resources from others. Common strategies included wearing pads, changing fluid intake patterns, planning routes, and planning for restroom locations.

EWA provides a suitable lens to investigate urinary concerns among older adults. With this lens, we have been able to describe the challenges caused by urinary concerns and the corresponding strategies that older adults use to adapt to them [3]. This also enabled us to gain a holistic understanding of resources that older adults seek and use [13]. Participants’ feedback indicated that that they had widely engaged with aging in their daily lives subconsciously, highlighting EWA’s value. In using the EWA framework, we as researchers and clinicians can see common patterns of changes in older adults’ behaviors and emotions, so that we can design programs to help them address challenges related to age-related changes that include but are not limited to urinary concerns.

By engaging with aging, older adults were not only able to address their urinary concerns but also achieved a sense of accomplishment and independence as a byproduct. Among half of the 29 older adults in our parent study, urinary concerns were a common issue. Furthermore, one third of our participants identified it as their primary concern, necessitating a need for adaptations. This finding corresponds with data presented by the American Urological Association in 2023, indicating that approximately 25–33% of older adults in the U.S. experience urinary problems [21].

Urinary concerns cause significant challenges for older adults who wish to maintain a stable lifestyle. One of their most prominent impacts among this study’s participants consisted of sleeping disturbances. Two recent studies have found a significant correlation between the prevalence of urinary concerns and heightened sleep disturbance and diminished sleep quality in older men and women [22, 23]. Other inconveniences mentioned by our participants included the inability to stay in places without restrooms for extended periods, increased efforts to plan routes, and the need to pinpoint restroom locations ahead of time. These findings are consistent with prior research on older adults with urge incontinence [7, 24], a condition in which older adults struggle to retain urine for extended durations and encounter challenges in looking for access to restrooms. Access to public restrooms has been significantly impeded during the COVID-19 pandemic, leading to a detrimental effect on quality of life for older adults who suffer from urinary incontinence. This might indicate policymakers’ neglect with respect to the needs of vulnerable older adults.

The finding that participants were inclined to make adaptations on their own rather than ask for support from family members or friends might indicate that their symptoms were not severe, given that all were community-dwelling individuals capable of independently performing activities of daily living. On the other hand, the self-sufficiency of elderly individuals in handling their urinary concerns may suggest a stigma related to openly discussing this condition with friends or family members [25]. Our results indicate that older adults would try to figure out a solution on their own before reaching out for help, and this is consistent with prior research [26–28].

The older adults in this study all responded that they could manage their urinary concerns at a satisfactory level. Their common strategies included changes in dietary patterns (e.g., fluid intake and food selection, taking supplements), medication schedules (e.g., times for taking diuretics), daily routines (e.g., planning routes and restroom locations, pelvic exercises), seeking help from healthcare (e.g., regular checkups, appointments, consultations), and utilization of tools (e.g. reminders, pads) and technologies (e.g., the virtual assistant Alexa). Various recent studies have suggested strategies to improve the management of urinary incontinence among community-dwelling older individuals. These strategies encompass conservative approaches [29], lifestyle adjustments (controlling the consumption of food, drugs, and fluids, pelvic exercises, and restriction of outdoor activities) [29–31], considering the use of physical devices (e.g., technology such as virtual assistants) [32], tools (e.g., absorbent pads and multilayer towels or cloths, wearing extra pants, and using public toilets), medications [29], and seeking guidance from healthcare professionals [27]. However, some of the strategies mentioned such as limiting water intake, for example, could be concerning from the perspective of healthcare. Dehydration is a prevalent and severe health concern among older adults [33]. Researchers and healthcare providers should help older adults revise their adaptation strategies as needed.

### Limitations and future directions

One limitation of this study consists of our sample’s demographics. The participants were primarily older adults with the technological capability to engage in virtual interviews via Zoom because of the pandemic, and this led to a group who were generally more educated. Although we know that qualitative findings are less intended for generalizability [34], future research may be needed to validate the generalizability of our findings to populations of less health literacy or lower socio-economic status. Future research may also consider incorporating strategies to improve participants’ diversity. Additionally, although we did include a male interviewer, the majority of interviewers were female, a factor that might have influenced male participants’ openness to share details about sensitive urinary symptoms. To address this potential bias, the interviewers built rapport as they proceeded through the interview process, and they used open-ended questions as well as prompts to help participants open up and disclose their personal experiences. As a result, we did not observe occasions of embarrassment or hesitancy in sharing among any participants during the interviews.

## Conclusion

Urinary symptoms, a prevalent outcome of age-related changes, often remain concealed because of associated emotions of shame and frustration. Most interviewees in this study reported being self-content about managing their urinary symptoms. Many encountered challenges in identifying suitable management strategies, resulting in protracted trial and error. Nonetheless, over time, participants managed to develop adaptive mechanisms that addressed their urinary concerns, which they eventually incorporated into their daily routines.

Healthcare professionals can play a pivotal role in fostering open dialogues about urinary concerns, potentially expediting the identification of tailored management strategies grounded in the etiology of patients’ issues. This approach holds promise for alleviating the emotional distress experienced by older adults who struggle with urinary symptoms and enhancing their overall quality of life. This qualitative study contributes to an understanding of emotional responses, adaptation processes, attitudes, encountered challenges, and adaptive outcomes in the context of urinary concerns among older adults. Insights from this research can inform targeted health education tailored to this specific demographic.

## Data Availability

All the qualitative data generated during this study are included in this published article. The datasets generated and/or analyzed during the current study are not publicly available due to the fact that the data is qualitative and has been thoroughly presented in the main text, but are available from the corresponding author on reasonable request.

## Declarations

### Clinical trial number

This study is not a clinical trial and a clinical trial number is not available.
